# Nondestructive Evaluation of Carbon Fiber Bicycle Frames Using Infrared Thermography

**DOI:** 10.3390/s17112679

**Published:** 2017-11-20

**Authors:** Rubén Usamentiaga, Clemente Ibarra-Castanedo, Matthieu Klein, Xavier Maldague, Jeroen Peeters, Alvaro Sanchez-Beato

**Affiliations:** 1 Department of Computer Science and Engineering, University of Oviedo, 33204 Gijón, Asturias, Spain; 2 Computer Vision and Systems Laboratory, Laval University, Quebec City, QC G1V 0A6, Canada; clemente.ibarra-castanedo@gel.ulaval.ca (C.I.-C.); xavier.maldague@gel.ulaval.ca (X.M.); 3 Visiooimage Inc., Infrared Thermography Testing Systems, 2604, Rue Lapointe, Quebec City, QC G1W 1A8, Canada; research@visiooimage.com; 4 Op3Mech Research Group, University of Antwerp, Groenenborgerlaan 171, B-2020 Antwerp, Belgium; Jeroen.peeters2@uantwerpen.be; 5 Actia Digital Ventures SRLU (THEBIKESPLACE.COM), 28413 Madrid, Spain; alvarosb@thebikesplace.com

**Keywords:** optical active infrared inspection, carbon fibre bicycle frame, nondestructive evaluation

## Abstract

Bicycle frames made of carbon fibre are extremely popular for high-performance cycling due to the stiffness-to-weight ratio, which enables greater power transfer. However, products manufactured using carbon fibre are sensitive to impact damage. Therefore, intelligent nondestructive evaluation is a required step to prevent failures and ensure a secure usage of the bicycle. This work proposes an inspection method based on active thermography, a proven technique successfully applied to other materials. Different configurations for the inspection are tested, including power and heating time. Moreover, experiments are applied to a real bicycle frame with generated impact damage of different energies. Tests show excellent results, detecting the generated damage during the inspection. When the results are combined with advanced image post-processing methods, the SNR is greatly increased, and the size and localization of the defects are clearly visible in the images.

## 1. Introduction

Nondestructive evaluation (NDE) is nowadays a fundamental technology to determine the quality and investigate the integrity of materials without damaging them. Nondestructive tests are used to detect defects, but also to prevent failures in order to ensure safe long-term operation [1]. Major innovations have been carried out in this field in recent decades, leading to important competitive advantages.

Many different techniques have been proposed for NDE. The most important can be classified as mechanical and optical, penetrating radiation, electromagnetic and electric, sonic and ultrasonic, thermal and infrared, chemical and analytical, image generation and signal and image analysis [2]. Infrared evaluation, in particular, has been proven to provide outstanding advantages compared to other techniques [3]. Infrared evaluation is fast, which enables high-speed scanning and major savings in time and cost. In addition, it is safe and suitable for prolonged and repeated use, with no harmful radiation effects such as in X-ray evaluation. Moreover, infrared evaluation is a non-invasive technique, where the inspected material is not affected or altered in any way [4].

NDE using infrared thermography is based on the acquisition and analysis of temperature and heat flow in the inspected material in order to detect subsurface anomalies. The most common approach for NDE is active infrared thermography. In this evaluation method, an external thermal stimulus is applied to the inspected material using optical flash lamps, heat lamps or other devices [5]. The thermal waves penetrate the surface of the material, producing a thermal contrast in areas with subsurface anomalies during the transient phase, which makes subsurface defect detection possible. The most common stimulation methods are pulse thermography, step heating thermography, lock-in thermography and ultrasound thermography. These methods can provide different results based on the material and the type of defect. NDE using active infrared thermography has been applied successfully to different materials, including carbon fibre-reinforced composites [6], steel [7], aluminium [8], walls [9], concrete [10] or cotton fibres [11], just to name a few.

One material with vast potential in many different industries is carbon fibre-reinforced polymer (CFRP), which is a composite material reinforced by carbon fibres. This material has excellent mechanical properties, including the modulus of elasticity and strength. Moreover, the material is not only strong, but also very lightweight. Additionally, it has far superior fatigue properties and corrosion resistance than metals, when combined with the proper resins [12]. Therefore, it is used in the manufacturing of numerous products where all these properties are of particular interest, such as the aerospace industry. The use of this material in other industries is still limited due to the cost, although in recent years, it has become more affordable. New high-performance products are taking advantage of CFRP’s superior features, such as sporting goods or automobiles. One of these new products is CFRP bicycle frames, which have become the standard material in the performance cycling world during the last decade, overtaking steel, aluminium and titanium.

CFRP is the most popular material for high-performance cycling due to the stiffness-to-weight ratio. CFRP bicycle frames are not only light; they are also extremely stiff, which enables greater power transfer. However, carbon fibre-derived products are also comparatively brittle, susceptible to damage caused by low energy impact loading, during manufacturing and also during service [13]. Low energy impact loading can create cracks and delamination due to the propagation of the mechanical energy inside the material, causing extensive subsurface damage invisible on the surface [14]. Numerous scenarios can provoke impact damage in a CFRP bicycle frame: a bicycle may fall and hit the floor; a small stone on the road can be projected by a nearby car; or simply due to tight clamps used for transportation. This type of damage in CFRP bicycle frames can lead to severe consequences: the frame can break, exposing the rider to a probable injury or even death [15]. On the surface, the bicycle frame may seem flawless, but the internal and hidden damage can provoke a very sudden and catastrophic failure. This is why the evaluation and inspection of CFRP bicycle frames are extremely important.

Different techniques have been applied to the nondestructive evaluation of CFRP bicycle frames [16], including pulse thermography, radiography, ultrasonic, acoustic and tap testing. However, although techniques to predict, detect and quantify impact damage in composite materials have been already studied [17,18,19,20], defect detection sensors for CFRP bicycle frames have not been analysed with rigour. This work proposes a novel inspection system for impact damage detection based on active infrared thermography. The proposed inspection method is fast, and it can be used to inspect a bicycle frame in seconds. It only requires an infrared camera and a source of heat for the thermal stimulus of the material. The resulting evaluation procedure is an effective method to detect damage in the bicycle frame. The proposed method is applied to real CFRP bicycle frames with generated impact damage of different energies to test the detectability and characterization of defects. Advanced image post-processing techniques are applied to the acquired data, and the results are evaluated using the signal-to-noise (SNR) metric. This work demonstrates the feasibility of the inspection procedure using an inexpensive sub-20k Euros camera and provides clear guidelines for an adequate configuration for a thermal stimulus and post-processing algorithm that greatly enhances the SNR.

Compared with other techniques, X-ray inspection could provide images where subsurface anomalies could be appreciated much more clearly than thermography. However, X-ray inspection is much more expensive, and there are serious radiation hazards for the technicians [21]. Ultrasound inspection could also be applied. Although, due to the complexity of the shape of a bicycle frame, many spots would be difficult to reach with the ultrasound heads. Besides, most of the defects occur very close to the surface, where ultrasound inspection is less accurate. Tap testing can be used as a complementary method on CFRP bikes. A tap test consists of gently tapping the area under inspection with a small hammer, listening for significant changes in sound. Dullness can indicate delaminations, but it requires a highly skilled operator to be a cost-effective method. Infrared thermography definitely offers interesting compromises for any CFRP inspection: it is fast, contact-less and efficient for a thin material like CFRP. This work demonstrates the feasibility of the inspection based on infrared thermography, with a very easy-to-apply method and good results.

The remainder of this paper is organized as follows. Section 2 presents the experimental design to evaluate the detectability of impact damage in CFRP bicycle frames; Section 3 discusses the results obtained with real data and image post-processing techniques; and finally, Section 4 reports the conclusions.

## 2. Experimental Investigation

### 2.1. Description of the Specimen

**Figure 1:**
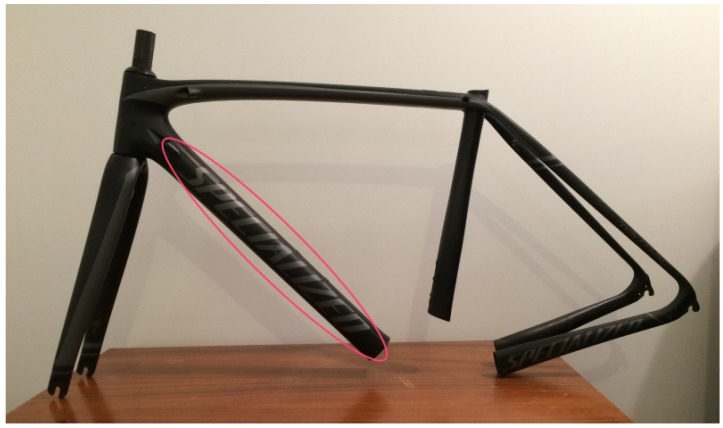
Inspected bicycle frame.

The bicycle frame used in the experiments has been manufactured by Specialized^TM^, one the most popular high-performance bicycle brands. Figure 1 shows an image of the inspected carbon fibre bicycle frame. The materials used in this bicycle are common for a carbon fibre bicycle frame. Thus, the results in this work can be easily extrapolated to most bicycles made of CFRP.

The bicycle frame is made of carbon fibre with reinforcements in some areas where different tubes are connected. The thickness of the tubes in the bicycle frame varies from 1.4 to 1.8 mm due to the bending of the carbon fibre composite during manufacturing. Experiments were performed in the down tube (highlighted in the image), which is the tube that connects the head tube and the bottom bracket. This tube can be clearly identified in the image because it contains the name of the brand. This tube is very close to the front wheel, where stones from the road can be projected, causing impact damage.

### 2.2. Infrared Camera

The infrared camera is a fundamental part of infrared evaluation. The camera is used to record the infrared radiation resulting from the thermal stimulus. The infrared camera used in the experiments is a Flir A655sc. The camera has a 24.6-mm lens and a sensor with a resolution of 640 × 480. The temperature range is configurable within several available ranges. In the experiments, the range [−40, 140 ${}^{\circ }$C] was selected. The manufacturer reports measurement accuracy of ±2 K and sensitivity lower than 30 mK at 30 ${}^{\circ }$C. The long-wave infrared camera operates at 7.5 to 14 μm. The detector type used in the camera is an uncooled microbolometer. The complete technical specifications are given in Table 1.

Uncooled cameras are not as sensitive as cameras based on cooled detectors that usually work in the mid-wavelength infrared band. However, the price of uncooled cameras is much lower, and the maintenance is greatly reduced. Therefore, the inspection of the bicycle frame using an uncooled camera presents a more practical approach, as NDE sensors based on this type of camera could be manufactured much more easily.

### 2.3. Estimation of Emissivity

**Figure 2:**
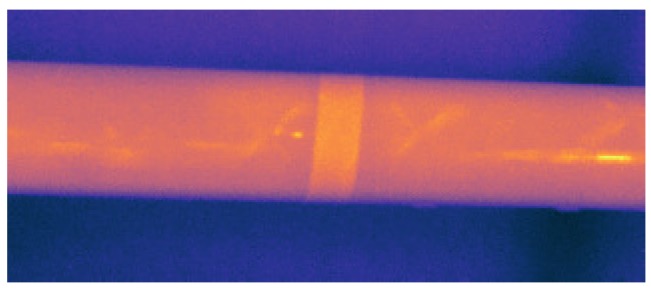
Infrared image of the experiment to measure emissivity using an electrical tape stuck on the surface.

The emissivity of the surface of the bicycle frame has been estimated using the reference emissivity material method [22]. Based on this method, the bicycle frame is heated with a piece of electrical tape with known emissivity stuck on the surface. The reference temperature on the electrical tape is used to measure the emissivity of the surface of the bicycle frame. Figure 2 shows an infrared image acquired during the experiment to measure emissivity using a thermal colour palette [23].

The result of the emissivity measurement procedure indicates that the global emissivity of the surface of the bicycle frame perpendicular to the camera is 0.82.

### 2.4. Impact Damage

**Figure 3:**
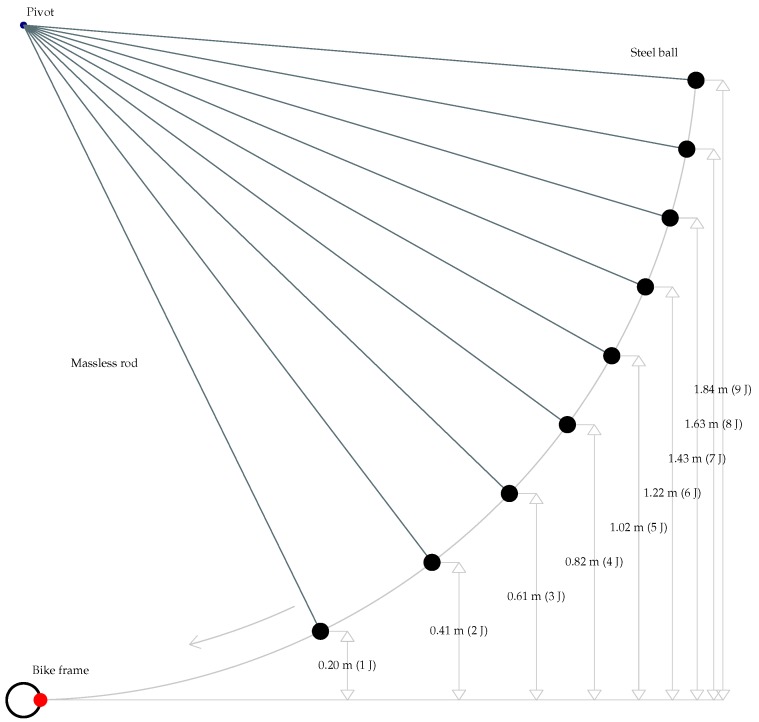
Procedure used to generate impact damage.

Impact damage is generated using a steel ball. The ball is designed and manufactured to perform the tests specified in different standards (IEC 60335, IEC 60065, IEC 60745, IEC 61029, IEC 60950). The ball is suspended from a pivot using a rod with negligible mass, creating a pendulum. The ball is displaced sideways from its equilibrium position with the rod fully extended. When released, the potential energy of the ball is transformed into kinetic energy, which is transferred to the bicycle frame during the impact. Figure 3 shows an illustration of the procedure to generate damage. The collision is considered elastic.

The energy of the impact depends on the mass of the ball, the height from the initial position to the impact position and the acceleration due to gravity. The ball used in the experiments is made of steel with a diameter of 50 mm and a mass of 0.5 kg. The acceleration due to gravity is also a constant. Thus, by suitably adjusting the height of the ball, different impact energies can be generated. As can be seen in the figure, when the ball is at 0.2 m, the energy is 1 J, and when the ball is at 1.84 m, the energy is 9 J. These are the ranges of impacts generated in the bicycle frame: from 1 to 9 J, increasing by 1 J.

**Figure 4:**
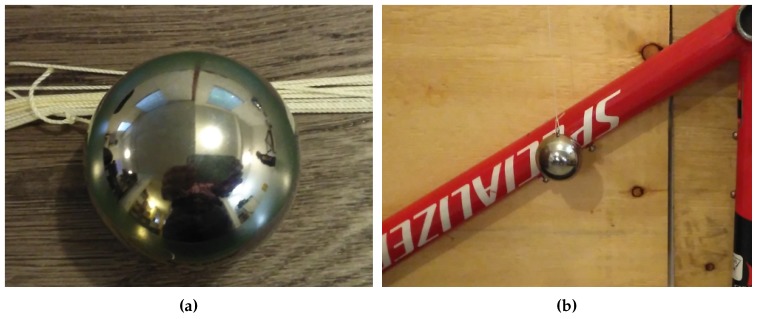
Calibrated steel ball used in the experiments. (**a**) Steel ball and rod. (**b**) Ball hitting a similar bicycle frame to produce impact damage.

Figure 4 shows the calibrated steel ball used in the experiments. The position of the bicycle frame was suitably adjusted so the ball hit the frame close to the centre of the tube.

The amount of damage generated in the bicycle frame is based on possible scenarios in which small stones in the road can be projected by a nearby car, the bicycle may fall and hit the floor or it simply has been generated during the manufacturing and assembly process. Accidents involving automobiles and bicycles can also be the source of impact damage. Damage can also result from abusive stress, overtightened devices and brackets over the carbon frame by users not respecting the manufacturer recommended torque or not using a torque wrench. In the case of mountain bikes, especially Enduro and Gravity (also known as downhill), there is a huge risk of impacts in the bottom bracket, downtube and chainstay. It is thus difficult to estimate what an average or typical defect on a carbon bike should be. Forces and areas of the damage can greatly vary, so can the resistance of the carbon frame for a given impact. CFRP thickness varies from 0.8 mm to 4 mm, with an average of 2 mm. Frame builders modify the thickness depending on the expected maximum load of each area. The idea proposed in this work is thus to cover a range of impacts from 1 to 9 joules, 1 joule being roughly the energy of impact of a standard full 33 cl aluminium can dropped from 30 cm height and 9 joules being the same can bottle dropped from about a 270-cm height.

**Figure 5:**
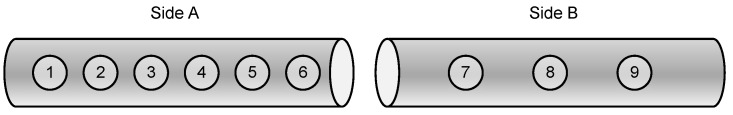
Map of defects on both side of the down tube and impact energy in joules.

The impact damage was generated on both sides of the down tube. On Side A, impacts of 1 to 6 J were generated. On the other side, Side B, impacts of 7 to 9 J were generated. Figure 5 illustrates the defect map on both sides of the down tube.

### 2.5. Infrared Inspection

In the infrared inspection, optical stimulation is applied to thermally stimulate the bicycle frame. Two halogen lamps are used. These lamps provide 1000 W at maximum capacity each. Two different configurations are used to compare the results: 1000 W at maximum capacity and 500 W at medium capacity. Therefore, in the first configuration, the bicycle frame is stimulated with a total of 2000 W and in the second with 1000 W.

The inspection method used in the experiments is usually referred to as optical step heating, or long or square pulse. Optical step heating uses a much longer pulse than optical pulse heating. The steps can last from a few seconds to a minute, and both the heating and the cooling responses are of interest. This type of inspection generates more heating than flashes, and it can be used to detect deeper defects. During heating or cooling, deviations from the temperature evolution of a sound area indicate subsurface anomalies or defects. Three configurations are used: 5-, 10- and 30-s pulses. After the lamps are turned off, the temperature decay is recorded for another 60 s, making a total of 65, 70 and 90 s. All experiments are performed with the camera operating at 50 Hz.

Considering the heating power with the halogen lamps (1000 and 2000 W), and the time they are turned on (5, 10 and 30 s), a total of 6 different configurations is applied to the inspected of each side of the frame.

**Figure 6:**
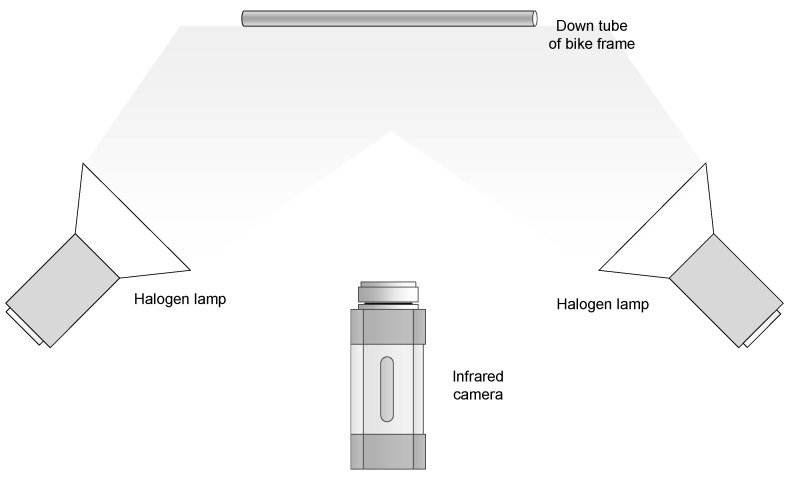
Infrared inspection in reflection mode.

The experiments are performed in reflection, i.e., the halogen lamps and the infrared camera are on the same side. This is the most appropriate configuration when the defects are close to the surface. Figure 6 shows an illustration of the configuration used. As can be seen, halogen lamps are positioned at both sides of the camera, and the inspected tube of the bicycle frame is in the middle, receiving the stimulus from the two halogen lamps.

**Figure 7:**
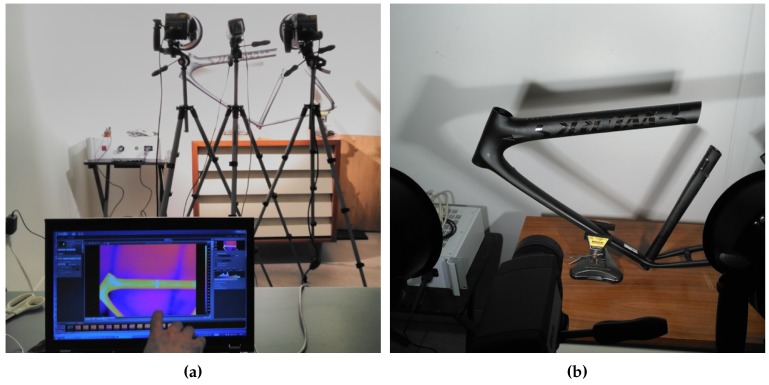
Inspection of the bicycle frame. (**a**) Complete view. (**b**) View from the camera position.

Figure 7 shows two images documenting the experimental setup. The bicycle frame was slightly rotated backward to reduce the reflections, as they can degrade the acquired infrared radiation by the camera. The camera and the halogen lamps are slightly tilted downwards as well to reduce the reflections of the halogen lamps on the bicycle frame.

### 2.6. Post-Processing

The defect contrast caused by the thermal stimulus in infrared evaluation can be very subtle, almost inappreciable in some cases. Therefore, the signal levels associated with subsurface anomalies can be lost in the data noise. In those cases, the defects are undetectable from raw thermograms. Post-processing methods are algorithms that improve the visualization and the contrast of the defects. Moreover, these algorithms tend to remove harmful artefacts such as non-uniform illumination from the images, increasing the signal-to-noise ratio and greatly improving the defect detection rate. The post-processing methods used in this work are briefly described next.

#### 2.6.1. Pulsed Phase Thermography

Pulsed phase thermography (PPT) is based on the calculation of FFT applied to the temperature-time history of every pixel in the acquired thermographic sequence. This operation approximates the temperature-time history by a sum of harmonic waves at different frequencies. The result of this operation describes the frequency response of the thermal stimulus. The operation is calculated using (1), where *i* is the imaginary number, *n* is the frequency increment and $Re_{n}$ and $Im_{n}$ are the real and imaginary parts of the DFT (discrete Fourier transform). The phase is of particular interest, as it contains relevant information about the structure of the material. It can be calculated using (2).

(1) $$F_{n}=\sum_{k=1}^{N-1}T\left(k\right)e^{\frac{2\pi ikn}{N}}=Re_{n}+Im_{n}$$

(2) $$\emptyset_{n}=\mathrm{atan}\left(\frac{Im_{n}}{Re_{n}}\right)$$

This method was originally proposed for pulse thermography [24], although it can also be applied to step heating inspections.

#### 2.6.2. Principal Component Thermography

Principal component thermography (PCT) is based on principal component analysis, a statistical technique of information synthesis. The goal is to reduce the number of variables in a dataset, while losing the least amount of relevant information possible.

The calculation of PCT is based on singular value decomposition (SVD), which decomposes the thermal sequence into a series of statistic orthogonal functions known as empirical orthogonal functions (EOF) [25]. The first components provide a reduction of the data without removing useful information about the defects.

#### 2.6.3. Polynomial Fit and Time Derivatives

In this method, the temperature-time history of every pixel in the thermal sequence is approximated by a polynomial. This method is usually called thermographic signal reconstruction (TSR) [26]. The evolution of the temperature is adjusted to an *n* degree polynomial as shown in (3).

(3) $$T\left(t\right)=a_{n}t^{n}+a_{n-1}t^{n-1}+\ldots +a_{1}t+a_{0}$$

When this method is applied to pulse thermography, the signals are previously converted to the logarithmic domain. However, this conversion is not required when applying this method to step heating thermography [27].

The polynomial fitting provides the opportunity to filter noise and compress the thermal sequence, as only the coefficients of the polynomials are required to reconstruct the sequence. Moreover, it can also be used to calculate the time derivatives analytically. These derivatives have been proven to be one of the best methods to enhance the visualization of defects [28], especially the second derivative.

#### 2.6.4. Partial Least Squares Thermography

Partial least squares thermography (PLST) is based on statistical correlation for the optimization of infrared inspection. The method decomposes the temperature-time history into a set of latent variables using partial least squares regression. In this method, non-relevant information is discarded, and only the most significant data are used in the regression. Non-uniform heating is removed while preserving the physical consistency [29].

## 3. Results and Discussion

### 3.1. Quantitative Evaluation

The analysis of the results in this work follows a quantitative approach. Therefore, it avoids subjective evaluation of the resulting images. In order to evaluate the results, the SNR metric is used to quantitatively assess the signal-to-noise ratio of the defects in the bicycle frames. The quantification of the defects is based on the definition of two regions in the images: the defect region and the reference or sound region [30]. The defect region encloses pixels in the images where the defect appears. This area is considered the signal. The sound region is an area in the image close to the defects, but outside the impact damage. This area is considered the noise. This approach to select the sound region close to the defect is usually referred to as the self-referencing method. This method is also recommended by the ASTM standards [31].

Prior information available about the defects is related to the approximate position in the bicycle frame. Thus, in this work, the defect and sound regions are calculated from the images obtained after the inspection. Some points in the images are selected as seed points. These points are placed in the centre of the defects. Using the intensity of these points as a reference, a similarity image is obtained using (4), where $I_{\mathrm{Ref}}$ is the intensity of the pixel selected as the seed. The resulting similarity image is then segmented using the fast marching method [32]. The results of the segmentation are the defect regions.

(4) $$J=\frac{1}{\sqrt{I-I_{\mathrm{Ref}}}}$$

The regions for the sound region are estimated around the defects using morphological dilatation applied to the binary image of the defects. Dilation is an operation used in mathematical morphology to expand the shapes of binary images. Two dilation operations are performed in the defect regions: a dilatation applied to the region of the defect to calculate a transitory region and a dilatation applied to this transitory region to calculate an extended region. The difference between these two regions is considered the reference or sound region. The diameter of the structuring element used in the morphological operation is the square root of the area of the region where the operation is applied, which follows the recommendation by ASTM [31].

**Figure 8:**
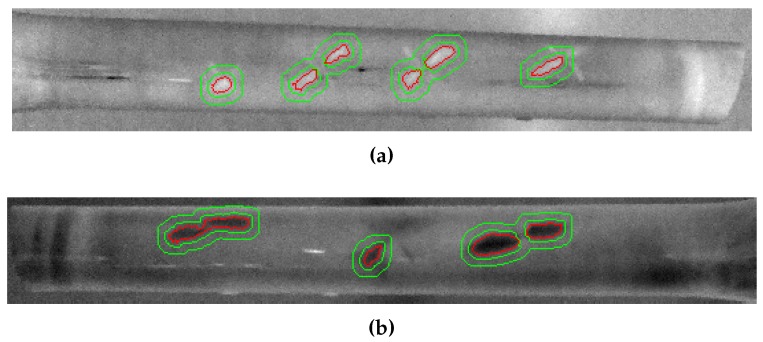
Defect and sound regions for the quantitative evaluation of the results. (**a**) Side A (from left to right, impact damage of 3, 4, 5 and 6 J). (**b**) Side B (from left to right, impact damage of 7, 8 and 9 J).

Figure 8 shows the obtained regions using the proposed procedure. The region in red is the defect region, and the region in green is the sound region. In this case, an image obtained with good contrast of the defects is used as a reference.

The SNR metric for the defects is calculated using (5), where $\mu_{S}$ is the arithmetic mean of all the pixels inside the defective area (signal), $\mu_{N}$ is the arithmetic mean of all the pixels inside the reference or sound area (noise) and $\sigma_{N}$ is the standard deviation of the pixels inside the reference area. This is not the only definition of SNR, but it is widely used in infrared inspection [28,33].

(5) $$\mathrm{SNR}=10\log_{10}\frac{{\left|\mu_{S}-\mu_{N}\right|}^{2}}{\sigma_{N}^{2}}$$

### 3.2. Analysed Periods

**Figure 9:**
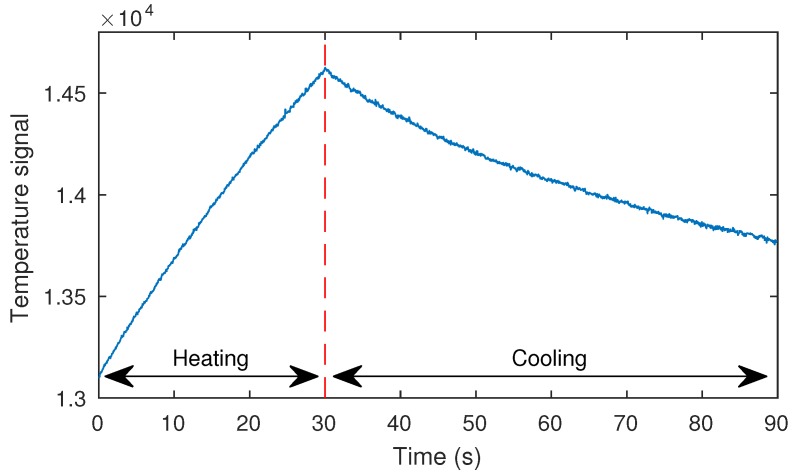
Periods of time considered for the analysis.

Before applying the post-processing methods, it is necessary to define the periods of interest from the infrared inspections. Two periods are considered in the optical step heating stimulation: heating and cooling. The periods are represented in Figure 9, which shows the temperature evolution of a pixel in the image after the thermal stimulus. In this case, a 30-s pulse configuration was used. The images in these two periods are extracted and analysed independently. The number of images extracted from the cooling period is always the same. However, in the heating period, the number of images depends on the time of the pulse.

### 3.3. Comparative Results

**Figure 10:**
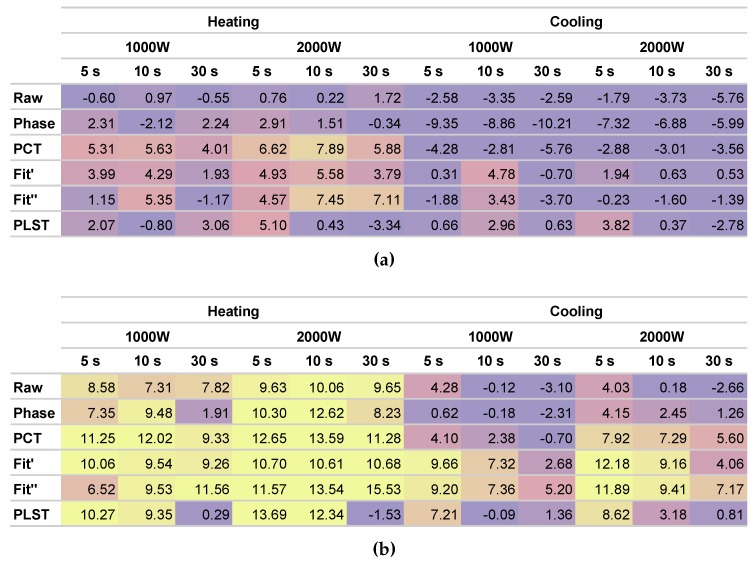
Quantitative results of the inspection for the considered periods, power, stimulation time and post-processing methods. All values are expressed in dB. (**a**) Side A with impact damage from 1 to 6 J. (**b**) Side B with impact damage from 7 to 9 J. SNR calculated as the average of all the impacts from each side.

The results of the experiments can be seen in Figure 10. The figure uses a colour scale to indicate the level of SNR: bright colours indicate high SNR and dark colours low SNR.

The results of the experiments show a huge difference between both sides of the bicycle frame. As expected, on Side B, the SNR is much higher because the impact energy of the defects is also higher. Thus, there is a correlation between SNR and impact energy. The SNR is higher as the energy of impact damage is increased, which makes defect detection easier.

The results also indicate that the heating period provides better information about the defects than the cooling period. In all the considered configurations (5-, 10- and 30-s pulses), the SNR results when processing the heating period are higher than the SNR results during cooling. Moreover, because the number of images during heating is significantly lower, the processing times are greatly reduced when processing the heating period. For example, in the 5-s pulse, only 250 images need to be processed. This is an important advantage, as the total time required to inspect the whole bicycle frame is reduced notably, providing the opportunity to design a fast nondestructive sensor.

There is a difference between applying a thermal stimulus of 1000 and 2000 W between 10 and 20%. The SNR improves as more energy is applied to the bicycle frame. A 2000-W stimulus seems to provide the best output, resulting in a high SNR. However, a 1000-W stimulus seems to be enough to detect the defects caused by impact damage in the bicycle frame.

Comparing the results of the three heating configurations (5-, 10- and 30-s pulses), it can be seen that the SNR results improve from a 5- to a 10-s pulse. However, in general, the results when the pulse is increased to 30 s are slightly worse. This indicates that the defects are very superficial, and the thermal contrast is generated at the beginning of the thermal stimulus. A 10-s pulse provides the best results, also with a reduced number of images to process.

**Figure 11:**
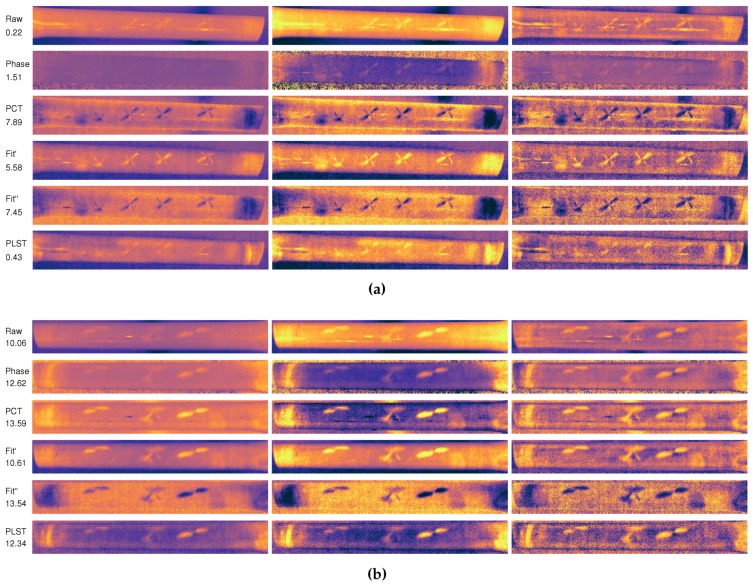
Images resulting from the infrared inspection during heating using a 10-second pulse and 2000 W. (**a**) Side A with impact damage from 1 to 6 J. (**b**) Side B with impact damage from 7 to 9 J.

Figure 11 shows a comparison between the images obtained from the infrared inspection during heating, using a 10-s pulse and 2000 W. The first column shows the image where the best SNR was obtained for the raw sequence and the sequences resulting from image post-processing. The second and third columns show a contrast enhancement of the first image. The second column shows an image where the histogram has been stretched removing the intensity values below 2% and 98%. The third column shows the results of applying a contrast-limited adaptive histogram equalization [34]. In these images, most of the impacts can be clearly distinguished. The only impacts that cannot be identified are impacts with 1 and 2 J. All the others can be clearly appreciated in the images, especially defects with high impact energy. As can be seen, the pattern of defects is similar to a butterfly wings pattern, where the direction is affected by the alignment of the fibres in the composite used for manufacturing the bicycle frame. This pattern is commonly found in infrared inspections of impact damage [3,35,36].

Low energy impacts (1 and 2 J) are not detected in the resulting infrared inspection. Those low energy impacts may have simply not created any damage, in which case it is normal to not detect them, or the limit of detection was reached using this specific setup. These type of impacts may be detected using infrared cameras with higher sensitivity. However, further exploration should be performed to analyse the detectability and consequences of this type of impact in the bicycle frames, or if they are causing any integral damage at all.

The results in Figure 10 and the images in Figure 11 also show a huge difference between the raw sequence and the results of the post-processing methods. The raw images have a very low SNR, while the post-processing methods greatly increase the resulting SNR, providing images where the defects can be appreciated easily. Therefore, the selected post-processing methods are helpful in improving the visualization and localization of the defects caused by impact damage.

All the post-processing methods tend to increase the SNR of the results. On average, the PCT method provides the best results, slightly above the second derivative of the polynomial fit. On the other hand, PLST provided the worse results, with irregular output across the experiments.

PCT also has a major advantage when compared with the other methods: the best SNR was always obtained in the third EOF, i.e., the third image in the resulting sequence of the PCT. This is an important advantage for inspection, as the technician can analyse this image without having to look at the entire sequence of images, as is normally the case with the polynomial fitting and derivatives.

**Figure 12:**
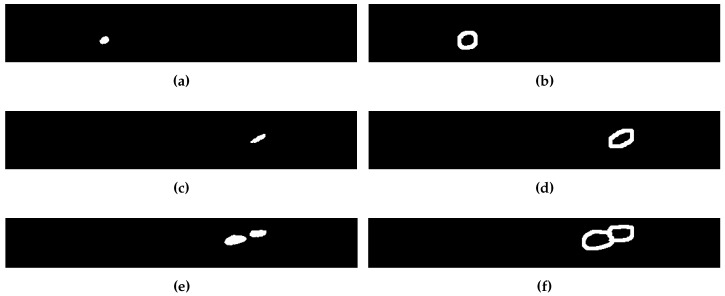
Defect and sound regions for defects with impact damage of 3, 6 and 9 J. (**a**) Defect region for 3 J impact. (**b**) Sound region for 3 J impact. (**c**) Defect region for 6 J impact. (**d**) Sound region for 3 J impact. (**e**) Defect region for 9 J impact. (**f**) Sound region for 9 J impact.

The calculated SNR in these results is an average of the SNR in all defects for each side. In order to evaluate the SNR of single defects, new regions for the defects and for the sound areas have been defined using the same procedure described above. Figure 12 shows the binary images of the defect and sound regions for the defects with impact damage of 3, 6 and 9 J.

**Figure 13:**
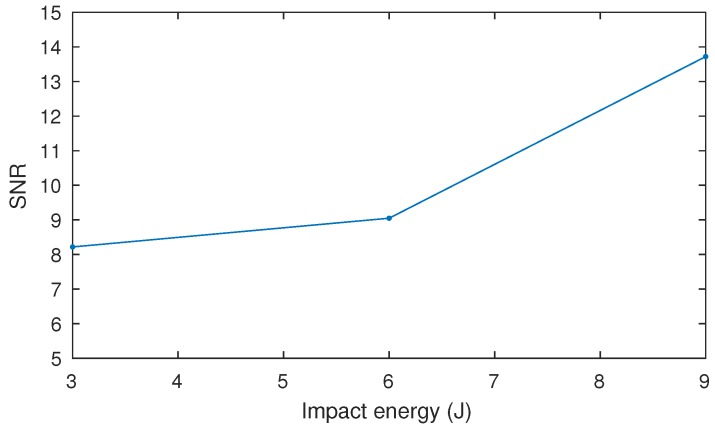
SNR for the there considered defects with impact energy of 3, 6 and 9 J.

**Figure 14:**
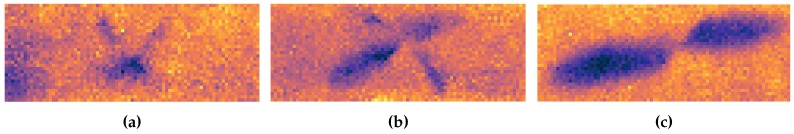
Images for the there considered defects. (**a**) Defect with 3 J impact energy. (**b**) Defect with 6 J impact energy. (**c**) Defect with 9 J impact energy.

The SNR for these three defects is represented in Figure 13, which indicates that the SNR increases with the impact energy. This result is consistent with previous works on the topic, which indicated that the intensity in the images increases with the impact energy [14]. Figure 14 shows the images of these three defects. The images are on the same scale. Thus, this figure can be used to compare the size of the defects. The area of the defects is another feature that is correlated with the impact energy: defects caused by high impact energy are larger. The particular pattern of impact damage can be clearly observed in these images.

## 4. Conclusions

Carbon-fibre composites are starting to be used in new and a wide variety of products. The resulting high-performance products are strong and also lightweight. However, these products are susceptible to damage caused by low energy impact loading not easily detected by visual inspection. Therefore, nondestructive evaluation is required in order to ensure safe long-term operation. High-performance carbon fibre bicycle frames are one of these products. In this case, impact damage can lead to dramatic consequences, exposing the rider to serious injury.

This work proposes a procedure to inspect carbon-fibre bicycle frames based on active infrared thermography. The proposed sensor is based on two halogen lamps and an infrared camera. The halogen lamps stimulate the bicycle frame thermally, and the infrared camera records the response. Different configurations have been tested for the inspection of the bicycle frames, including the power of the lamps and the time during which the lamps were turned on. These configurations were applied to a set a generated impact damage in a real carbon-fibre bicycle frame. The results were analysed quantitatively, including advanced image post-processing methods to improve the visualization of the localization of the defects.

The results indicate that lamps with a power of 1000 W turned on during only 5 s provide very good detection results. Most defects were clearly appreciable in the resulting infrared images. Moreover, when post-processing methods were applied, the SNR was greatly increased. The optimal configuration was a stimulation energy of 2000 W during 10 s. When combining the infrared sequence resulting from this configuration with principal component thermography, optimal results were obtained in terms of SNR. The resulting images clearly showed the size and intensity of the defects, which could be used to infer the impact energy of the damage.

The proposed inspection procedure in this work can be used to evaluate a carbon-fibre bicycle frame very quickly without damaging it. A similar approach is very likely to find potential applications in a number of different areas, where new products manufactured using carbon-fibre composites are starting to become more popular.

## Figures and Tables

**Table 1:** Technical specifications of the infrared camera FLIR A655sc used in the experiments.

Camera	FLIR A655sc
Temperature range	−40 to +140 ${}^{\circ }$C
Thermal sensitivity/Noise Equivalent Temperature Difference (NETD)	30 mK at 30 ${}^{\circ }$C
Detector	640 × 480 UFPA
Spectral range	7.5–14 μm
Image frequency	50 Hz
Spatial resolution	0.68 mrad
Field of view (FOV)	25${}^{\circ }$ × 19${}^{\circ }$
Detector pitch	17 μm
